# Betanodavirus infection in primary neuron cultures from sole

**DOI:** 10.1186/s13567-018-0580-4

**Published:** 2018-09-05

**Authors:** Sandra Souto, José G. Olveira, Lucía Vázquez-Salgado, Carlos P. Dopazo, Isabel Bandín

**Affiliations:** Departamento de Microbiología y Parasitología, Instituto de Acuicultura, Universidade de Santiago de Compostela, 15706 Santiago de Compostela, Spain

## Abstract

Nervous necrosis virus (NNV), G. *Betanodavirus*, is the causative agent of viral encephalopathy and retinopathy, a disease that causes mass mortalities in a wide range of fish species. Betanodaviruses are neurotropic viruses and their replication in the susceptible fish species seems to be almost entirely restricted to nerve tissue. However, none of the cell lines used for NNV propagation has a nervous origin. In this study, first we established a protocol for the primary culture of neurons from Senegalese sole, which made it possible to further study virus-host cell interactions. Then, we compared the replication of three NNV strains with different genotypes (SJNNV, RGNNV and a RGNNV/SJNNV reassortant strain) in sole neuron primary cultures and E-11 cells. In addition, to study how two amino acid substitutions at the c-terminal of the capsid protein (positions 247 and 270) affect the binding to cell receptors, a recombinant strain was also tested. The results show that sole neural cells enabled replication of all the tested NNV strains. However, the recombinant strain shows a clearly delayed replication when compared with the wt strain. This delay was not observed in virus replicating in E-11 cells, suggesting a viral interaction with different cell receptors. The establishment of a sole primary neuronal culture protocol provides an important tool for research into betanodavirus infection in sole.

## Introduction

Viral encephalopathy and retinopathy (VER), otherwise known as viral nervous necrosis (VNN), is a highly infective neuropathological condition that affects a wide range of fish species worldwide and causes mass mortalities, mainly in larval and juvenile stages [1]. The causative agents are Nervous necrosis viruses (NNV), small and non-enveloped viruses belonging to the G. *Betanodavirus*, F. *Nodaviridae*. The betanodavirus genome consists of two single stranded RNA molecules, RNA1 (3.1 Kb) and RNA2 (1.4 Kb), which encode the RNA-dependent RNA polymerase and the capsid protein, respectively [2]. A subgenomic transcript, known as RNA3, is synthesized during RNA replication from the 3′ terminus of RNA1. Betanodaviruses have been classified into four genotypes: striped jack nervous necrosis virus (SJNNV), tiger puffer nervous necrosis virus (TPNNV), redspotted grouper nervous necrosis virus (RGNNV) and barfin flounder nervous necrosis virus (BFNNV) [3], based on a small variable sequence of RNA2, the so-called T4 region. However, the isolation of reassortant strains between RGNNV and SJNNV genotypes has been reported from Senegalese sole (*Solea senegalensis*), gilthead sea bream (*Sparus aurata*) and sea bass (*Dicentrarchus labrax*) in southern Europe [4–7]. Reassortant strains isolated from diseased farmed Senegalese sole and gilthead sea bream in the Iberian Peninsula showed an RNA1 typed as RGNNV and an SJNNV-type RNA2 and exhibited a modified SJNNV capsid amino acid sequence [4]. We previously demonstrated that two of these modified positions (residues 247 and 270) play a major role in betanodavirus virulence for Senegalese sole, possibly due to a slower spread in fish brain tissue [8].

Betanodaviruses have a clear neurotropism [9] and their replication in the susceptible fish species seems to be almost entirely restricted to nerve tissue, preferentially the brain and retina [10, 11]. Although different fish cell lines, including RTG-2, CHSE-214, BF2, SBL, FHM and EPC, have been tested for susceptibility to NNV [2, 12–15], the first successful isolation of a betanodavirus was achieved using the SSN-1 cell line, established from whole fry tissue of striped snakehead *Ophicephalus striatus* [16]. Subsequently, the GF-1 cell-line derived from grouper *Epinephelus coioides*, E-11, a clonal line derived from SSN-1 cells, and SB derived from Asian sea bass *Lates calcarifer* was also demonstrated to be useful for the isolation and proliferation of NNV [17–19]. It has been suggested that viral replication in these cell-lines and earlier failure in established cell lines may be due to the existence of a specific receptor for NNV [17]. However, none of these cell-lines has a neuronal origin and may not present specific receptors, such as the neural cell adhesion molecule (NCAM), a cell adhesion glycoprotein that in the rabies virus, a well-known neurotropic virus, plays a role in entry [20]. In the present study, we established a protocol to obtain primary neuron cultures from sole and analysed the effect of the two capsid mutations observed in the reassortant strains on viral replication in the neural cells.

## Materials and methods

### Experimental animals

Senegalese sole (20–50 g) were obtained from commercial hatcheries and kept at the aquarium facilities of the University of Santiago de Compostela at 22 °C. Upon arrival, some fish were sacrificed with an anaesthetic overdose (MS-222, tricaine methane sulphonate, Sigma) and used for diagnosis of bacterial pathogens as well as regular viral agents, including infectious pancreatic necrosis virus (IPNV), infectious haematopoietic necrosis virus (IHNV), viral haemorrhagic septicaemia virus (VHSV) and betanodavirus as described by [21]. All efforts were made to minimize the number of animals used and their suffering.

### Isolation of brain cells

Senegalese sole were euthanized by an overdose of the anaesthetic MS-222. Fish were sprayed and wiped using 70% ethanol, and their brains were removed aseptically from the skull and immersed in Hank’s buffer (Lonza) supplemented with 2 mM glucose and 200 µg/mL gentamicin (dissection medium). Tissues were washed three times with the dissection medium and then placed in pools of 5 brains in a clean Petri dish with fresh medium and minced using a scalpel into smaller sections of 2–3 mm. The primary culture of the isolated tissue was undertaken using enzymatic disaggregation by incubating the tissues in 6 mL of Neurobasal medium (Gibco) supplemented with 2 mM glutamine (isolation medium) with a proteolytic enzyme. The first attempts to isolate neural cells were done using two different enzymes, papain and trypsin to assess their performance. Half of the tissue sample was incubated with a 20 U/mL papain solution (Sigma) for 30 min at 30 °C in a shaking water bath and the other half was incubated with 0.1% trypsin (Lonza) for 15 min at room temperature (RT). After allowing non-dispersed tissue to settle, the enzymes were removed and 2 mL of fresh medium were added. Then, the tissue was triturated with a flame-polished Pasteur pipette for 1 min. After allowing non-triturated tissue to settle for 1 min, the supernatant was transferred to an empty 15-mL tube. This procedure was repeated twice combining all the supernatants from each sample. Subsequently, the cell suspension was carefully applied to the top of a prepared OptiPrep density gradient as described in [22]. The gradient was centrifuged at 800 × *g* for 15 min at 22 °C. The top 6 mL containing cellular debris was discarded whereas three different fractions were collected separately; the top 1 mL of the gradient (Fraction 1), enriched for oligodendrocytes; the following 1 mL (Fraction 2) containing cell fragments, neurons and other cells, and the 2 mL at the bottom excluding the pellet (Fraction 3) enriched for neurons. Cell fractions 1 and 2 were discharged and fraction 3 was diluted with 10 mL isolation medium and centrifuged at 200 ×* g* for 2 min at 22 °C. The supernatant was discarded and the cells were washed once more. The pellet was resuspended in 1 mL of the culture medium (see below) and the number of cells was estimated. Viability was tested using trypan blue dye exclusion. Cells were plated at a concentration of 2 × 10^5^ cells/cm^2^ in pre-coated 0.5 mg/mL poly-d-lysine (Sigma) 24-well plates for primary cultures (Sarstedt). Two different growth media were tested: Dulbecco’s Modified Eagle Medium with Nutrient Mixture F-12 (DMEM/F12, Gibco) and Leibovitz’s L-15 Medium. Both media supplemented with 1× B-27 (Gibco), 15% FBS (Gibco), 2 mM glutamine (Lonza), 15 ng/mL basic fibroblast growth factor (bFGF, Sigma) and 100 µg/mL gentamicin. After 24 h, the media was partially removed and the wells were refilled with fresh culture media. To investigate the influence of temperature on cell proliferation, the sole brain cells were cultured at 15, 20, 25 and 30 °C. Cultures were examined daily and graded for confluency.

### Indirect immunolabeling

Immunolabeling with a neuronal marker was used to identify neural cells. The medium from cells grown on coverslips was removed and cells were fixed for 20 min at −20 °C in a solution of acetone:ethanol (1:1). Subsequently, the cells were washed three times for 5 min in PBS/Tween 0.05%. The cells were incubated with the primary antibody against neurofilaments (NF-200, Sigma) at room temperature (RT) for 1 h and washed three times in PBS/Tween 0.05%. The coverslips were then treated with FITC-conjugated anti-mouse immunoglobulins (Sigma) in PBS/Tween 0.05% for 1 h at RT in the dark. Then they were washed again three times for 5 min in PBS/Tween 0.05% and stained with DAPI (Sigma) according to the manufacturer’s instructions. Immunofluorescence-labelled cells were analysed using a Nikon fluorescence microscope.

### Betanodavirus replication in sole brain cells

#### Viruses, cells, and virus growth

The betanodavirus strains used in this study were the following: SGWak97 and SJNag93 belonging to the RGNNV and SJNNV genotypes, respectively [23] SpSs-IAusc160.03, a reassortant RGNNV/SJNNV strain isolated from diseased farmed sole [4], hereafter wild-type strain or wt160 and the recombinant strains rSs160.03 and rSs160_247 + 270_ previously described in detail [8], hereafter r160 and r247 + 270, respectively. These two recombinant strains have been produced by reverse genetics; r160 has a genome sequence identical to wt160, whereas r247 + 270 bear two amino acid substitutions at the C-terminal of the coat protein and has lower in vivo fitness than wt160 [8]. The viruses were maintained and propagated in E-11 cells [17] with L-15 medium containing 2% foetal bovine serum (FBS), penicillin (100 IU/mL) and streptomycin (100 μg/mL) at 25 °C and stored at −80 °C, as previously described [8]. Viral strains were titrated in triplicate by the endpoint dilution method on 96-well plates. The TCID_50_/mL (tissue-culture infectious dose infecting 50% of inoculated cultures) was calculated according to the method described in [24].

#### Infection of brain cells with betanodavirus

Three well plates containing sole brain cells (70% confluence) approximately 15 days post-seeding were infected with either wt160, recombinant viruses, SGWak97 or SJNag93, at a M.O.I of 0.01. After 1 h adsorption, the virus was removed and 1 mL of fresh medium L-15 supplemented with 1× B-27 (Gibco), 2% FBS (Gibco), 2 mM glutamine (Lonza) and 100 µg/mL gentamicin was added and incubated at 25 °C. Three non-infected wells per plate were used as negative controls. Samples from supernatant (0.1 mL) were collected at various times post-infection (pi): 1, 5, 7, 9, 11 and 12 days post-infection (dpi) and an equal amount of fresh medium was subsequently added to the wells at each time point. Virus yields were determined by RT-qPCR.

#### Infection of E-11 cells with betanodavirus

Three well plates containing E-11 cells were infected with the same strains described above at an M.O.I of 0.01. After 1 h adsorption, the virus was removed and 1 mL of fresh medium L-15 supplemented with 2% foetal bovine serum (FBS), penicillin (100 IU/mL) and streptomycin (100 μg/mL) was added and incubated at 25 °C. Samples from supernatant were collected at: 1, 3, 5 and 6 dpi and subjected to RT-qPCR.

### Virus quantification by RT-qPCR

RNA was extracted from 0.1 mL of the cell supernatants using the EZNA Total RNA kit (Omega Bio-tek), according to the manufacturer’s instructions. The total RNA collected was suspended in 50 µL molecular-grade water and stored at −80 °C. cDNA synthesis was performed by mixing RNA with random primers, heating at 95 °C for 5 min and incubating at 4 °C for at least 1 min. Then, a reverse transcription mixture containing Superscript III RT (Invitrogen) was added and incubated at 25 °C for 10 min. RT reactions were performed at 50 °C for 50 min, followed by 5 min at 85 °C for RT enzyme inactivation. The cDNA was then added into real-time PCR buffer, which included 0.2 mM forward and reverse primers SnodR1 [21] in 1× NZYTaq Green Master Mix (NZYTech). The quantitative PCR was carried out using the CFX96TM Real-Time PCR Detection System (Bio-Rad) as previously described [8] with a thermocycling program consisting of an initial denaturation/activation step at 95 °C for 15 min, and 40 cycles of amplification (denaturation at 95 °C for 15 s, annealing and extension at 60 °C for 15 s). To prepare the standard curve, 20-fold dilutions of a plasmid containing the full-length RNA1 of strain SpSs-IAusc160.03 were prepared. Viral load data were calculated as RNA1 copies per mL of supernatant. All samples were tested in triplicate.

### Statistics

Statistical analyses of all data were performed using GraphPad Prism 5.0. All results obtained from quantitative RT-PCR were expressed as mean ± SD. Data were tested for significance by analysis of variance (two-way Anova), followed by Bonferroni post hoc tests to determine differences among the obtained virus concentrations. Mean values were considered significant when *p* < 0.05.

## Results

The first aim of this study was to establish the optimal conditions for primary cell culture from the brain tissue of Senegalese sole and subsequently test their susceptibility to betanodavirus infection.

### Optimal culture conditions establishment

The isolation and culture of the brain cells was carried out following the protocol described by [22] for the isolation of neurons and neurospheres from adult rats or mice with some modifications. Different conditions were tested in order to obtain the most suitable protocol for the sole neuron culture development and maintenance.

Initial investigations into the best method to disaggregate tissue demonstrated that papain digestion resulted in a higher yield of cells than trypsin. Digestion using papain led to a complete disaggregation of the tissue, a higher viability of the harvested cells and also a better cell attachment after seeding.

The OptiPrep density gradient made it possible to isolate purer cell fractions, reducing the presence of cellular debris and blood cells. Three different fractions were initially used to produce the primary cultures. Neuron and neurosphere development was higher when using fraction 3 compared to fractions 1 and 2, as indicated by the immunolabeling of the anti-neurofilament neuronal marker. Therefore, this fraction was used for all subsequent experiments.

To determine the more suitable temperature conditions after the isolation protocol, neural cells were incubated at 15, 20, 25 and 30 °C. Survival and confluency varied depending on temperature. The lowest viability was obtained at 15 and 30 °C. Poor attachment of the cells and a low rate of cellular differentiation were observed at 15 °C, while at 30 °C cells were able to attach at the beginning and axons began to develop but then necrotic cells appeared after 4–6 days and most of the cell layer was detached from the well surface by days 7–8 post-seeding. The best cellular adhesion and cell differentiation was observed at 20 and 25 °C; even though the best temperature for growing was determined to be 25 °C, because at this temperature the cultures developed a more complex neuronal network.

The cultivation of cells in L-15 medium resulted in an increased survival of cells and neurite differentiation after seeding. L-15 enhanced neurons developed branching neurites forming a neuronal network, whereas wells cultured with DMEM/F12 show more proliferation of glial cells and nonneuronal cell types, such as fibroblasts. Moreover, after 2–3 days at 25 °C and without supplementation of exogenous CO_2_, the DMEM/F12 medium was not able to maintain the optimal pH conditions, becoming too basic and causing detrimental effects on the cells. Hence, L-15 medium was selected for further experiments.

**Figure 1:**
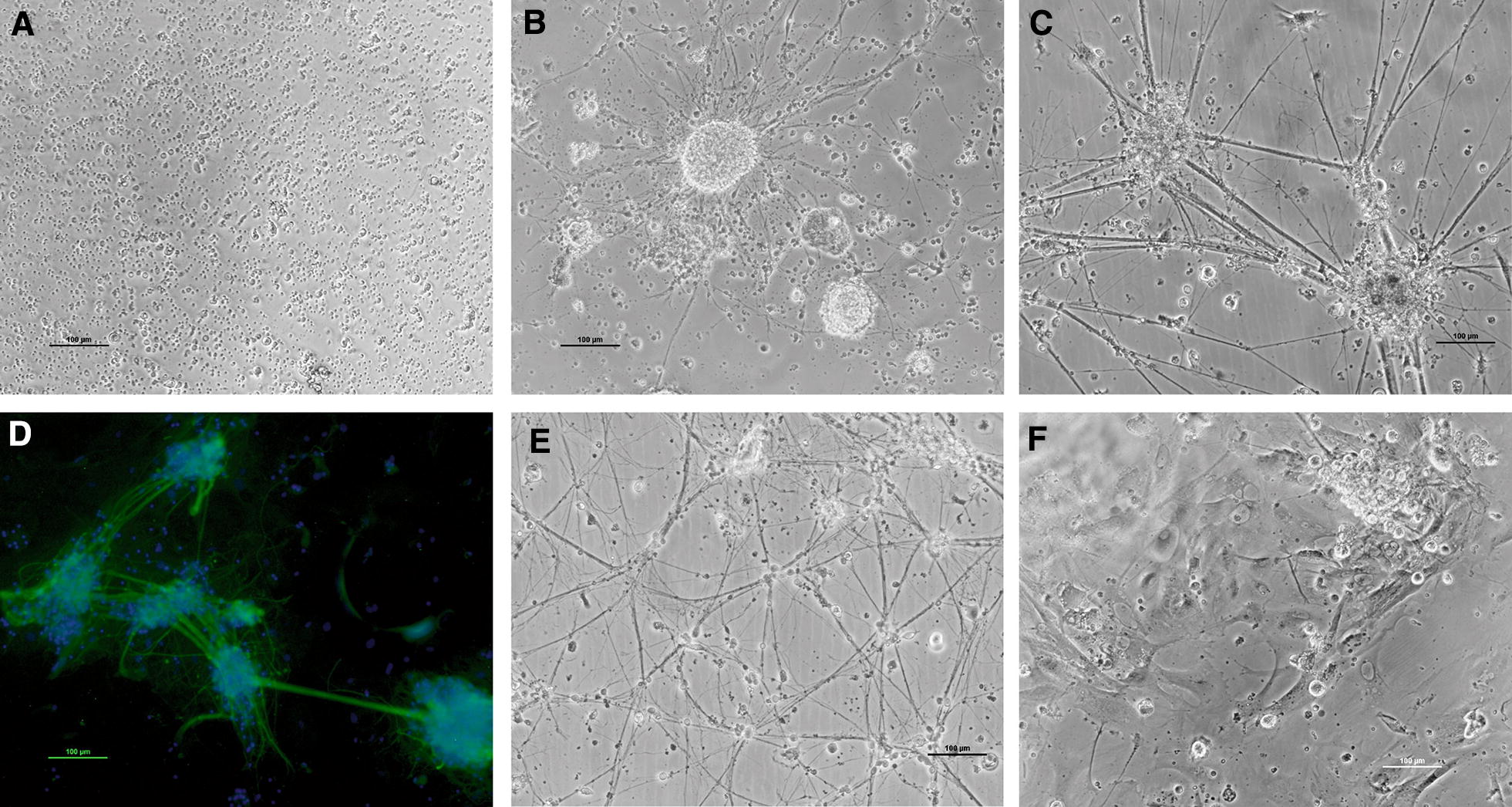
**Primary sole neuronal cultures. A** Spherical cells after 24 h, **B** cell aggregates, **C** neuronal connections, **D** immunofluorescent staining with the neurofilament specific antibody (NF-200), **E** differentiated cells after 15 days in culture, **F** differentiated cells after 30 days in culture. Scale bar 100 µm.

After 24 h, primary cultures, obtained with the optimum conditions described above, show cells with a spherical morphology (Figure 1A). By days 4–5, numerous bright cell aggregates with a spherical shape that did not attach to the well surface and that were identified as neurospheres, were observed (Figure 1B). Then, between days 5 and 15 post-seeding cells began to attach and proliferate, neurite-like fibers grew and contacted with neighbouring aggregates (Figure 1C). Immunocytochemical staining showed that the cells were marked with the neuron specific antibody NF-200 (Figure 1D). In addition, different types of cells proliferated to form a layer of adherent cells that developed to reach confluence under the neural cells. At day 15, the cultures reached the maximum neurite development (Figure 1E). Healthy primary cultures could be maintained for approximately 1 month (Figure 1F).

### Infection of brain cells with betanodavirus

**Figure 2:**
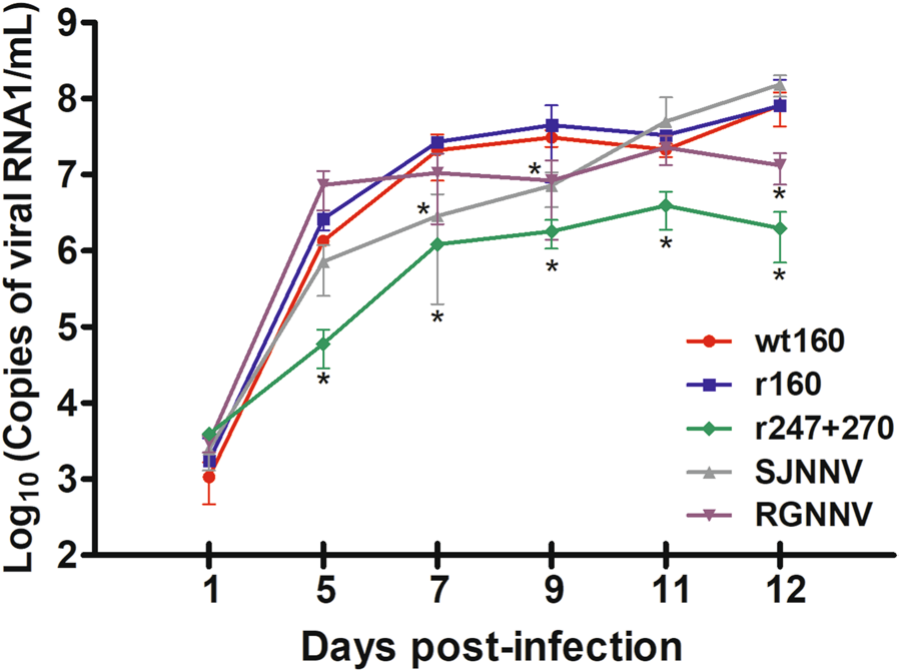
**Viral replication in brain cells from primary cultures.** Samples were analysed by RNA1 quantification by RT-qPCR per mL of supernatant. Means and standard deviations from three wells are presented. *p < 0.05 for differences with the wild type virus (wt160).

**Figure 3:**
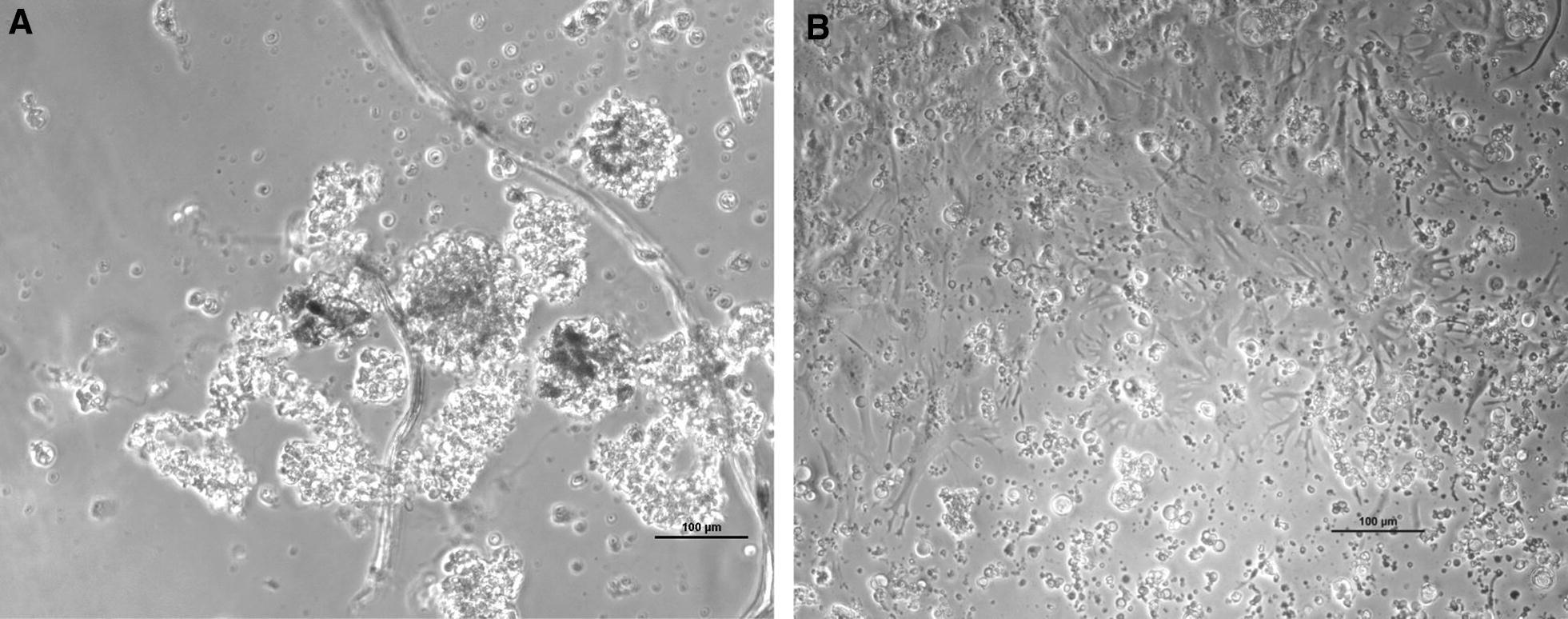
**Primary sole neuronal cultures after 28** **days in culture. A** CPE at 12 dpi with wt160, **B** control uninfected cells.

The replication in the neuron primary cultures of NNV strains belonging to different genotypes, including the recombinant strain r247 + 270, was evaluated by real-time qPCR. No virus was detected in the non- infected wells. All strains were able to enter cells and replicate; at day 1 pi all strains displayed a similar RNA1 load (1.1 − 3.9 × 10^3^ copies/mL) (Figure 2). The highest viral replication at 5 dpi was observed with the RGNNV strain SGwak97 (7.3 × 10^6^ copies/mL), the replication of this strain increased until day 9 but then reached a plateau. The SJNNV strain showed a different behaviour, a slow load increase from 5 to 9 dpi and a higher increase afterwards. Both, the wt160 and r160 strains show very similar replication kinetics, with a continuous increase throughout the whole experimental period and reaching maximum values of 8 × 10^8^ copies/mL at 12 dpi. When compared with the reassortant strain, SJNag93 and SGwak97 show a significant replication delay at days 7 and 9, respectively. Thereafter, the SJNNV strain reached values similar to those of the reassortant strain, whereas the RGNNV strain had a significantly lower RNA1 load (*p* < 0.05). It is interesting to note that the genomic load was significantly lower in r247 + 270 than in the wild type strain or r160. Sequence analysis of PCR products confirmed that mutations were maintained during replication in neural cells. Cell destruction (80–100%) was observed by day 12 pi in all the infected wells (Figure 3A), which were clearly differentiated from control cells (Figure 3B). Wells inoculated with the r247 + 270 strain presented a reduction in the severity of the cytopathic effect (CPE).

### Infection of E-11 cells with betanodavirus

**Figure 4:**
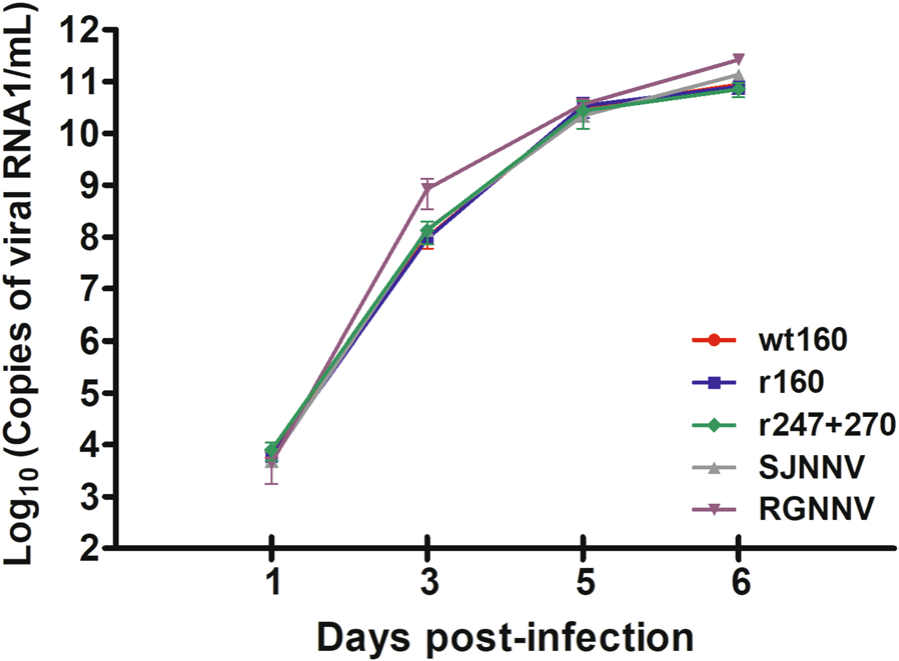
**Viral replication in E-11 cells.** Data are expressed as viral RNA1 detected in E-11 cell supernatants. Means and standard deviations from three wells are presented.

In the in vitro infection using E-11 cells, very similar replication kinetics were observed between the different strains (Figure 4). Although at day 3 pi the RNA1 copy number of SGWak97 was nearly 1 log higher than that of the reassortant strain, afterwards no significant differences were observed between the strains and they both reached very similar values at the end of the experiment (6 dpi when complete destruction of the cell monolayers was observed). Viral production in E-11 was 3 logs higher than in neurons for reassortant strain (wt and r160) and SJNag93, and 4 logs higher for SGwak97 and r247 + 270.

## Discussion

Betanodavirus replication in fish shows a marked preference for neurological tissues, mainly the brain and retina. We previously reported that reassortant strains RGNNV/SJNNV isolated from sole show two amino acid substitutions in the capsid protein with respect to the SJNNV capsid [4]. These substitutions seem to delay the virus spreading in sole brain tissue but do not affect viral replication in E-11 cells [8]. This different replication behaviour in vivo and in vitro prompted us to look for a neural cell culture to confirm the effect of capsid substitutions in betanodavirus replication in its target cells, namely neurons. However, although the establishment of a culture of stem neural cells from sea bass has been reported [25], due to technical problems and contamination, as indicated by the authors, these cells are not available to the scientific community. Therefore, in this study we established a protocol for isolation and primary culture of sole neurons and we assessed the replication of different NNV strains in these cells. In addition, we comparatively analysed the effect of capsid mutation on betanodavirus replication in the neural cells and the E-11 cell line.

Adult neurogenesis is a phenomenon found in the brain of all vertebrates studied to date. In teleost fish, neurons are produced in all brain subdivisions [26]. Different protocols have been published to obtain brain cells from different fish species [25, 27–31] and the most used enzymes to digest tissue samples are papain and trypsin. Therefore, to develop the sole neurons isolation protocol we evaluated the dispersion capacity of both enzymes. Papain treatment yielded the best results because a higher number of dissociated viable cells was obtained. In addition, these cells were better than those treated with trypsin in terms of development and attachment to the plate surface. These results concur with those obtained from the isolation of neurons from the tropical freshwater fish species *Hoplias malabaricus* [31]. As media composition and temperature have been reported to affect the growth of different brain cells [25, 29, 30, 32], the influence of both factors on sole neuron development was investigated. When DMEM/F12 was used, most new cells differentiated into glial cells, which almost covered all the plate surface, and few neurospheres and neurons were observed, thus the final neuron count was quite low. However, when L-15 was used, a proper neuronal network was set up achieving maximal growth at 15 days and healthy monolayers were maintained up to 30–35 days. Different results have been reported on the usefulness of each media to support brain cell grown. Whereas L-15 has also been successfully used for culturing neural stem cells from sea bass [30] or astrocytes from rainbow trout [29], DMEM/F12 has been reported to provide excellent results for the isolation of neurons from the tropical fish species *H. malabaricus* [31], *Apteronotus leptorhynchus* [25] and *Astatotilapia burtoni* [33]. Although four different growth temperatures were assayed, only two proved to be appropriate for sole neuron development. Low (15 °C) temperatures did not support the growth of sole brain cells, although this fish species can be found in the wild between 13 and 28 °C [34]. Temperatures lower than 20 °C have also been reported to be inadequate for rainbow trout astrocyte culture [29]. High temperatures (30 °C) did not allow successful growth either. However, at 20 °C and 25 °C a high number of neurospheres was obtained and good cellular adhesion and cell differentiation was observed; 25 °C was considered the best temperature because cell cultures developed a more complex neuronal network. Temperatures higher than 20 °C have also been chosen to culture sea bass neurons [30].

All five NNV strains were replicated in the sole neural cells and displayed a similar genomic load at day 1 pi. However, afterwards, the recombinant strain harbouring substitutions at positions 247 and 270 in the capsid protein showed a clearly delayed replication when compared with wt160 and r160. Both amino acids are located on the outer surface of the capsid [35, 36] and therefore may be involved in the interaction with the host cell surface. We previously postulated that these amino acidic changes may modify the affinity of virus for cellular receptors and affect its spread through sole brain [8] and the marked replication delay of the recombinant throughout our experiments supports this hypothesis. As previously reported, a single mutation in the capsid affects the alphavirus Sindbis virus binding to and spreading through neural cells [37]. It is interesting to note that the point mutations performed on r247 + 270 reverted the substitutions observed in the reassortant strains back to the SJNNV-type; but although SJNag93 (the SJNNV strain used in this study) showed a replication delay from days 5 to 9 with respect to the wt and r160 strains, thereafter it reached similar RNA values. This result indicates that other factors besides these capsid amino acids must be involved in the delayed replication of r247 + 270. Although changes in positions 247 and 270 are shared by all reassortant strains isolated in the Iberian Peninsula [4], the wt 160 strain shows three additional differences in the coat protein sequence from the SJNag93 strain, at positions 13, 20 and 79. These three changes are not in the C-terminal side of the coat protein and therefore it is not probable that they are involved in host cell interaction. However, we observed that the substitution of amino acid 20 causes a delay in the viral replication in the brain tissue of experimentally infected fish, which is increased if fish are infected with mutant harbouring substitutions at positions 20, 247 and 270 (unpublished results). Experiments are in progress to study the effect of all capsid substitutions on the reassortant replication in neural primary cultures.

On the contrary, the experiments of replication in E-11 cells, shorter in time than experiments with neural cells because of the viral destruction of the monolayers, showed almost identical results for all 5 strains, and no delay of the mutant strain was observed. In addition, a higher viral production than in neural cells was obtained, although this could be due to the different number of cells present in each culture. The different replication behaviour of r247 + 270 in both E-11 and sole neurons could be explained by an interaction with different cell receptors. Sialic acid seems to be involved in the binding of betanodavirus to SNN-1 cells [38] and therefore also to E-11, which are a clone of SSN-1 [17]. However, in other cell lines, other NNV receptors have been identified. Thus, in GF-1 grouper heat shock cognate protein 70 (GHSC70) has been proposed as an NNV receptor or co-receptor protein [39] and in SB cells receptors have been reported to probably be proteins located at lipid rafts or even specific lipids [40]. Although so far no data regarding the neuronal receptors involved in NNV entry is available, it is quite probable that neuronal receptors are different to those present in these cell lines, because two of them, SSN-1 and SB were derived from whole fry or larvae tissue [16, 41] and GF-1 from fin tissue [18]. In other viruses, like rabies viruses, it has been reported that most of the fixed rabies virus laboratory strains have acquired the ability to use ubiquitous receptors present at the surface of non-neuronal cell types, which are different from the neuronal receptors used to propagate in the nervous system [42].

In conclusion, although we have not been able to establish a long-term culture of sole neural cells, we have obtained primary cultures that lasted for a month and enable betanodavirus replication. NNV replication in neurons pointed out the existence of cell-receptors different to those characterized in E-11 cells, and probably in other cell lines.

## Abbreviations

NNV: nervous necrosis virus
SJNNV: striped jack nervous necrosis virus
RGNNV: redspotted grouper nervous necrosis virus
RTG-2: rainbow trout gonad cells
CHSE-214: Chinook salmon embryo cells
BF2: blue gill fibroblast cells
SBL: sea bass larvae cells
FHM: fathead minnow cells
EPC: epithelioma papulosum cyprini cells
SSN-1: striped snakehead cells
GF-1: grouper fin cells
SB: Asian sea bass fibroblast cells larvae
NCAM: neural cell adhesion molecule
bFGF: basic fibroblast growth factor
M.O.I: multiplicity of infection
NCR: non coding regions
pi: post-infection
CPE: cytopathic effect
